# *Spirulina platensis* Alleviated the Oxidative Damage in the Gills, Liver, and Kidney Organs of Nile Tilapia Intoxicated with Sodium Sulphate

**DOI:** 10.3390/ani10122423

**Published:** 2020-12-17

**Authors:** Eman M. Awed, Kadry M. Sadek, Magdy K. Soliman, Riad H. Khalil, Elsayed M. Younis, Abdel-Wahab A. Abdel-Warith, Hien Van Doan, Mahmoud A.O. Dawood, Hany M.R. Abdel-Latif

**Affiliations:** 1 Department of Biochemistry, Faculty of Veterinary Medicine, Damanhour University, Damanhour 22511, Egypt; ema.cloepatra@yahoo.com (E.M.A.); ksaadek@gmail.com (K.M.S.); 2 Department of Poultry and Fish Diseases, Faculty of Veterinary Medicine, Damanhour University, Damanhour 22511, Egypt; m.khalil@gmail.com; 3 Department of Poultry and Fish Diseases, Faculty of Veterinary Medicine, Alexandria University, Edfina 22758, Behera Province, Egypt; Riad.Khalil@alexu.edu.eg; 4 Department of Zoology, College of Science, King Saud University, P.O. Box 2455, Riyadh 11451, Saudi Arabia; emyounis@ksu.edu.sa (E.M.Y.); aaabdelwarith@yahoo.com (A.-W.A.A.-W.); 5 Department of Animal Production, Faculty of Agriculture, Al-Azhar University, Nasr City, Cairo 11651, Egypt; 6 Department of Animal and Aquatic Sciences, Faculty of Agriculture, Chiang Mai University, Chiang Mai 50200, Thailand; 7 Innoviative Agriculture Research Center, Faculty of Agriculture, Chiang Mai University, Chiang Mai 50200, Thailand; 8 Department of Animal Production, Faculty of Agriculture, Kafrelsheikh University, Kafrelsheikh 33516, Egypt

**Keywords:** oxidative stress, natural antioxidants, aquaculture, expression analysis, phytogenic

## Abstract

**Simple Summary:**

Nile tilapia (*Oreochromis niloticus*) are expected to suffer from oxidative stress induced by sodium sulphate in the ecosystem. Herein, we proposed that dietary *Spirulina platensis* could relieve the impacts of sodium sulphate on tilapia. The hepatic antioxidative and related activities were decreased under sodium sulphate exposure. However, dietary *S. platensis* alleviated the tissue antioxidative overexpression compared to the sodium sulphate and control groups. This study implies that natural dietary antioxidants can be applied in aquatic organisms to alleviate the features induced by toxicants and xenobiotics.

**Abstract:**

The current study aimed at assessing the recuperative roles of dietary *Spirulina platensis* on the antioxidation capacity of Nile tilapia (*Oreochromis niloticus*) exposed to sodium sulphate for eight weeks. In brief, fish were allocated into four groups with three triplicates per group, where a group fed on a commercial basal diet served as control, a group was intoxicated with sodium sulphate (SS) 5.8 mg/L, another group was fed a diet supplemented with 1% *S. platensis* (SP), and the last group was fed 1% SP and concomitantly intoxicated with 5.8 mg/L sodium sulphate (SP/SS). Tissue antioxidative indices of each fish were measured as follows: glutathione peroxidase (GSH-Px) activity in muscles, catalase (CAT) and superoxide dismutase (SOD) activities in gills, and total antioxidant capacity (T-AOC) in the liver and kidney. Moreover, the expression of hepatic SOD, GSH-Px, and glutathione-S-transferase (GST) genes was also determined. It was found that tissue CAT, SOD, and GSH-Px activities as well as the T-AOC levels were significantly decreased in the SS group (*p* < 0.05). Moreover, there was a significant downregulation of hepatic SOD, GSH-Px, and GST genes in SS-exposed fish (*p* < 0.05). Interestingly, simultaneous dietary supplementation with SP provided a marked attenuation of the tissue antioxidative parameters when compared with the SS and control groups. To conclude, the present study exemplifies that dietary SP supplementation could be a beneficial abrogation of SS-induced tissue oxidative stress in the exposed fish.

## 1. Introduction

Aquatic organisms may be exposed throughout their life to various toxicants and environmental pollutants, which may consequently affect their health, induce serious toxicological signs, and result in heavy mortalities [1,2]. From these pollutants, sulphates are naturally occurring in the aquatic environments following plant decomposition, atmospheric deposition, and human activities, such as mining [3]. Their concentrations can also be increased due to the discharges from agricultural runoff, treatment plants, and industrial processes, such as tanneries as well as paper and textile mills [3]. Previous studies reported that sodium sulphate (SS) induces toxic effects in exposed fathead minnows (*Pimephales promelas*) [4] and Nile tilapia (*Oreochromis niloticus*) [5]. The exposure of fish to environmental pollutants will induce oxidative stress because of the overproduction of reactive oxygen species (ROS), which will subsequently induce tissue damage and death [2,6,7].

Gills, muscles, liver, and kidney are the main organs that suffer from the toxicity with waterborne xenobiotics [6,8]. More specifically, the gills are responsible for respiration and osmoregulation, whereas the liver plays a vital role in the detoxification of toxins and xenobiotics in the fish body [9]. Meanwhile, the kidney secretes urine and the remaining salt derivatives produced during the metabolic functions in the fishes’ body [10,11]. Once the inflammatory features induced by oxidative stress and free radicals exist, these organs might dysfunction [12].

Herbal feed supplements are widely utilized in aquafeeds with unique and various benefits to fish [13,14,15]. *Spirulina platensis* (SP) is a filamentous blue-green microalga with potent immunostimulant, anti-inflammatory, and antioxidant activities because of its unique phytochemical contents [16]. Interestingly, Sayed et al. [17] illustrated that SP can activate a specific series of both biochemical and physiological activities to neutralize the toxic impacts of environmental chemicals in fish. In this regard, there are extensive studies that have shown the beneficial bioremediation effects of SP for attenuation of the toxicity signs of various toxicants in fish, such as aflatoxin B_1_ [12], insecticides [18], and heavy metals [19,20].

Globally, Nile tilapia is regarded as the second most cultured and consumed freshwater finfish species after carps and characterized by their successful rearing in tropical and subtropical conditions [21,22]. Furthermore, Nile tilapia is considered as an ideal example to study the effects of environmental pollutants on the aquatic ecosystem [23]. In light of SS toxicity and SP’s possibility to alleviate the oxidative stress induced by toxicants and xenobiotics as mentioned above, the present study proposed that dietary feeding with SP is expected to counteract oxidative stress resulting from SS toxicity for the first time. Herein, the present study focused on an evaluation of the recuperative efficacy of dietary SP on the tissue antioxidation capacity of SS-exposed Nile tilapia.

## 2. Materials and Methods

### 2.1. Ethical Statement

The protocol of the present experiment, and fish rearing techniques were approved (NCLS2000) and conducted with the guidance of the Research Ethical Committee, Faculty of Veterinary Medicine, Damanhour University, Egypt.

### 2.2. Fish and Rearing Conditions

Nile tilapia juveniles with an initial weight of 40.31 ± 3.0 g were purchased from a local farm in Kafr Elsheikh province, Egypt, and left for two weeks to be adapted to the laboratory conditions. Fish were randomly distributed in 12 glass aquaria (100 L) (70 × 50 × 60 cm) (10 fish per aquarium/4 groups/triplicates). The aquaria were filled with dechlorinated tap water and supplied with constant and continuous aeration using compressed air pumps. The light was maintained at 12:12 h light: dark cycle during the day by using fluorescent light tubes. Throughout the whole experimental period, to maintain clear water, 50% of the water in each aquarium was daily siphoned to get rid of feces and uneaten food and then was replenished with new well-aerated water from the storage tank. The water quality characteristics were analyzed weekly and the values of water physical and chemical parameters were maintained as follows: 7.6 ± 0.4 for pH, 0.02 ± 0.001 mg/L for the un-ionized ammonia NH_3_, 6.50 ± 0.4 mg/L for the dissolved oxygen (DO), 0.015 ± 0.001 mg/L for nitrite (NO_2_), and 25 ± 0.4 °C for water temperature according to Boyd and Tucker [24].

### 2.3. Experimental Setup

The basal diet was formulated to supply the nutritional requirements for optimal growth of Nile tilapia according to National Research Council (NRC) guidelines [25] (Table 1). All feed ingredients of the basal diet were thoroughly mixed with water and oil and then supplemented with 1% *Spirulina platensis* (SP) (Agent Chemical Laboratories, Redmond, WA, USA) and pelleted using a meat grinder. Fish were allocated in four groups with three replicates in each (10 fish/replicate) and fed the formulated diets for a successive eight weeks. Fish were fed the diets at a dose rate of 3% of their live body weight three times daily (9:00, 12:00, and 16:00 h). Sodium sulphate (SS) solution (Lab Service Co., Alexandria, Egypt) was prepared by diluting a stock solution with distal water. The final concentration of 5.8 mg/L was selected based on the previous study by Awed et al. [5]. The experimental groups were named as following:

G1: control group fed the basal diet only;
G2 (SS): fed the basal diet and intoxicated with waterborne SS 5.8 mg/L [5];
G3 (SP): fed a diet supplemented with 1% SP [26]; and
G4: fed a diet supplemented with 1% SP and intoxicated with waterborne SS 5.8 mg/L.

### 2.4. Sample Collection

At the end of the trial (8 weeks), all fish were fasted 24 h before the final sampling. Then, fish in each tank were weighed to calculate the growth performance indices. The following equations were used for the calculation: Weight gain (WG, %) = (FBW − IBW) × 100/IBW, specific growth rate (SGR, %g/day) = 100((LnFBW − LnIBW)/T), where FBW = body weight final (g), IBW = body weight initial (g), and T = duration (days).

Then, all fish were euthanized with a solution of tricaine methane sulphonate (MS-222) up to 400 mg/L. Parts of the gills, liver, muscles, and kidneys were then gently collected from three fish per aquarium (9 fish/group) on an ice-cold plate, washed with physiological cold saline, and dehydrated by filter paper.

### 2.5. Tissue Samples Preparation and the Measurement of the Antioxidative Indices

The collected liver, kidney, and gill tissues were homogenized in cool physiological buffer saline. The tissue homogenate was then filtered and centrifuged at 1500 rpm for 20 min at 4 °C using a fast-cooling rotator Type 3K-30, Sigma Laborzentrifugen GmbH, Osterode am Harz, Germany. The clear supernatant was assembled and then stored at −80 °C for use for spectrophotometric determination of superoxide dismutase (SOD), glutathione peroxidase (GSH-Px), catalase (CAT), and total antioxidant capacity (T-AOC) using specific commercially purchased diagnostic kits (Bio-diagnostics, Giza, Egypt). The CAT and SOD were evaluated in gills using the method of Aebi [27], and Nishikimi et al. [28], respectively. GSH-Px was assessed in the muscular tissue according to the method of Paglia and Valentine [29]; meanwhile, the T-AOC in liver and kidney tissues was evaluated according to Koracevic et al. [30].

### 2.6. Transcriptomic Expression Analysis of Antioxidant Genes

Total RNA was extracted from 100 mg of the fish hepatic tissue of three fish per aquarium (9 fish/group) using Trizol iNtRON (iNtRON Biotechnology, Inc., Seongnam, Gyeonggi, Korea) following the manufacturer’s manual. Confirmation of the quality and quantity of the extracted RNA was done using a Nanodrop Uv–Vis spectrophotometer Q5000/Quawell, San Jose, CA, USA). Next, the complementary DNA (cDNA) was synthesized using the SensiFAST™ cDNA synthesis kits, Bioline/Meridian Bioscience, London, UK) following the manufacturer’s protocols. The gene-specific primer sequences used for *SOD* [31], *GSH-Px* [32], glutathione-S-transferase (*GST*) [33] genes, and *β-actin* as a housekeeping gene [34] and their accession numbers in the NCBI GenBank are illustrated in Table 2.

The qRT-PCR Stratagene MX3000P was used for the evaluation of gene expression using the SYBR green method to quantify the gene expression folds (SensiFast SYBR Lo-Rox kit, Bioline/Meridian Bioscience, London, UK). The thermocycling conditions for the reaction were 95 °C for 30 s, followed by 45 cycles of denaturation at 63 °C for 60 s and annealing at 60 °C for 60 s. The mRNA expression folds of the examined antioxidant genes were standardized to *β-actin* according to the 2^−ΔΔCT^ method [35].

### 2.7. Statistical Analysis

All the data were analyzed by one-way analysis of variance ANOVA (SPSS^®^ version 22, SPSS Inc., Chicago, IL, USA), and followed by Duncan’s post-hoc test to compare the means of treatment groups. Data were expressed as means ± standard errors (SE) and the difference of treatment effects were considered significant at *p* < 0.05.

## 3. Results

The antioxidation capacity of Nile tilapia fed SP and exposed to sub-chronic toxicity with SS is shown by detecting the levels of GSH-Px in muscular tissues, CAT, and SOD in gills, and T-AOC in liver and kidney tissues. Moreover, the expression of antioxidant genes SOD, GST, and GSH-Px in the liver tissues also contributes to the antioxidative capacity of Nile tilapia.

### 3.1. Growth Performance

The growth performance of tilapia groups fed the basal diet with or without SP and exposed to SS is presented in Table 3. The FBW, WG, and SGR indices were not significantly affected by dietary SP with or without SS toxicity (*p* > 0.05).

### 3.2. Muscle, Gills, Liver, and Kidney Antioxidation Capacity

**Figure 1:**
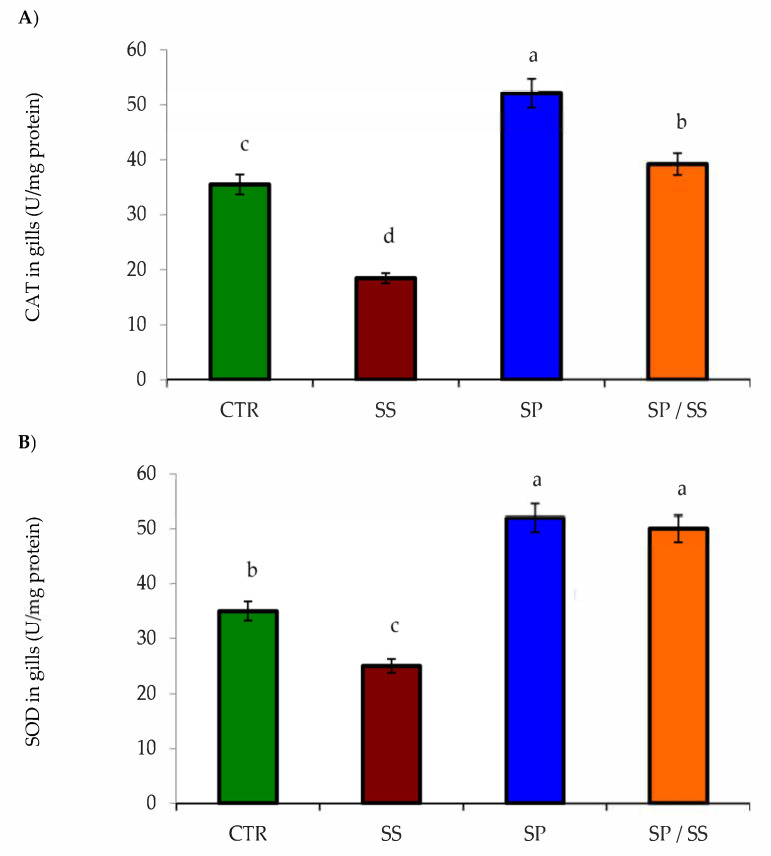
(**A**) Catalase (CAT) and (**B**) superoxide dismutase (SOD) levels in the gills of Nile tilapia after sodium sulphate (SS) exposure and fed diets with or without *S. platensis* (SP) for 8 weeks. Values are expressed as mean ± standard errors (SE) (*n* = 3). a,b,c,d = letters on top of the bars show that the groups are significantly different from those in the control group (CTR) (*p* < 0.05).

**Figure 2:**
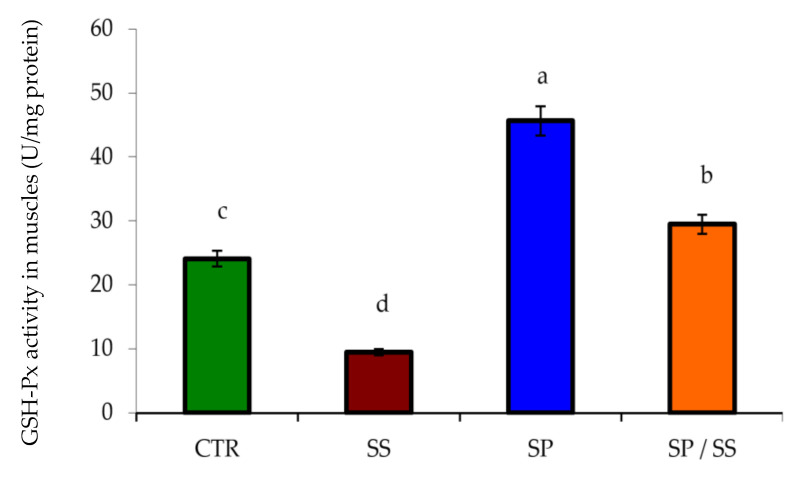
Glutathione peroxidase (GSH-Px) levels in muscle tissues of Nile tilapia after sodium sulphate (SS) exposure and fed diets with or without *S. platensis* (SP) for 8 weeks. Values are expressed as mean ± standard errors (SE) (*n* = 3). a,b,c,d = letters on top of the bars show that the groups are significantly different from those in the control group (CTR) (*p* < 0.05).

**Figure 3:**
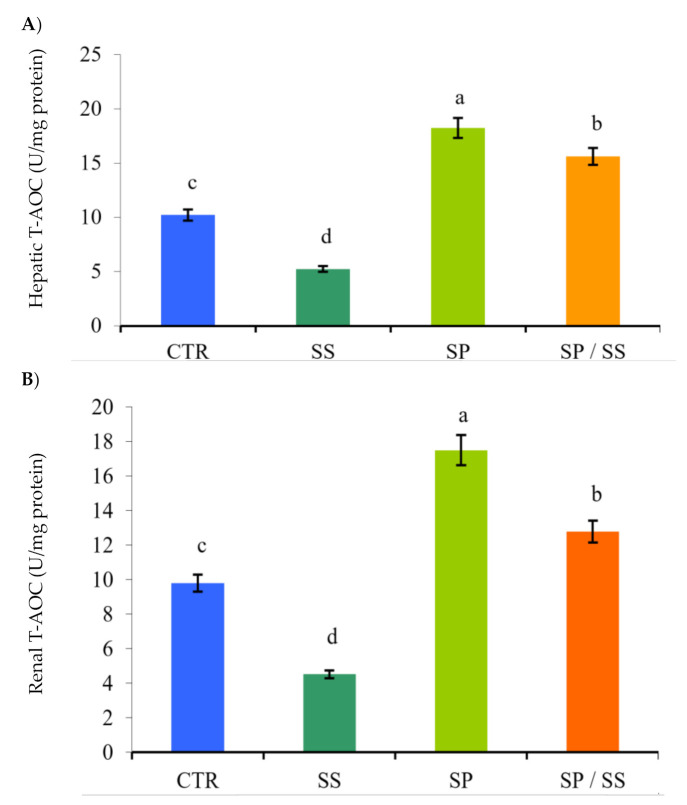
Total antioxidant capacity (T-AOC) levels in the liver (**A**) and kidney (**B**) tissues of Nile tilapia after sodium sulphate (SS) exposure and fed diets with or without *S. platensis* (SP) for 8 weeks. Values are expressed as mean ± standard errors (SE) (*n* = 3). a,b,c,d = letters on top of the bars show that the groups are significantly different from those in the control group (CTR) (*p* < 0.05).

The lowest values of gills CAT (Figure 1A), SOD (Figure 1B), GSH-Px (Figure 2), and T-AOC in the liver (Figure 3A) and T-AOC in the kidney (Figure 3B) were found in the SS group (*p* < 0.05) in comparison with other groups. Moreover, their values had the opposite trend regarding the SP-supplemented group. Interestingly, Nile tilapia fed the diet co-supplemented with SP and concomitantly exposed to SS for 8 weeks had significantly increased values when compared with the SS group (*p* < 0.05), and these results suggest a considerable attenuation of the oxidative stress induced in the SS group.

### 3.3. Expression of Hepatic Antioxidant Genes

**Figure 4:**
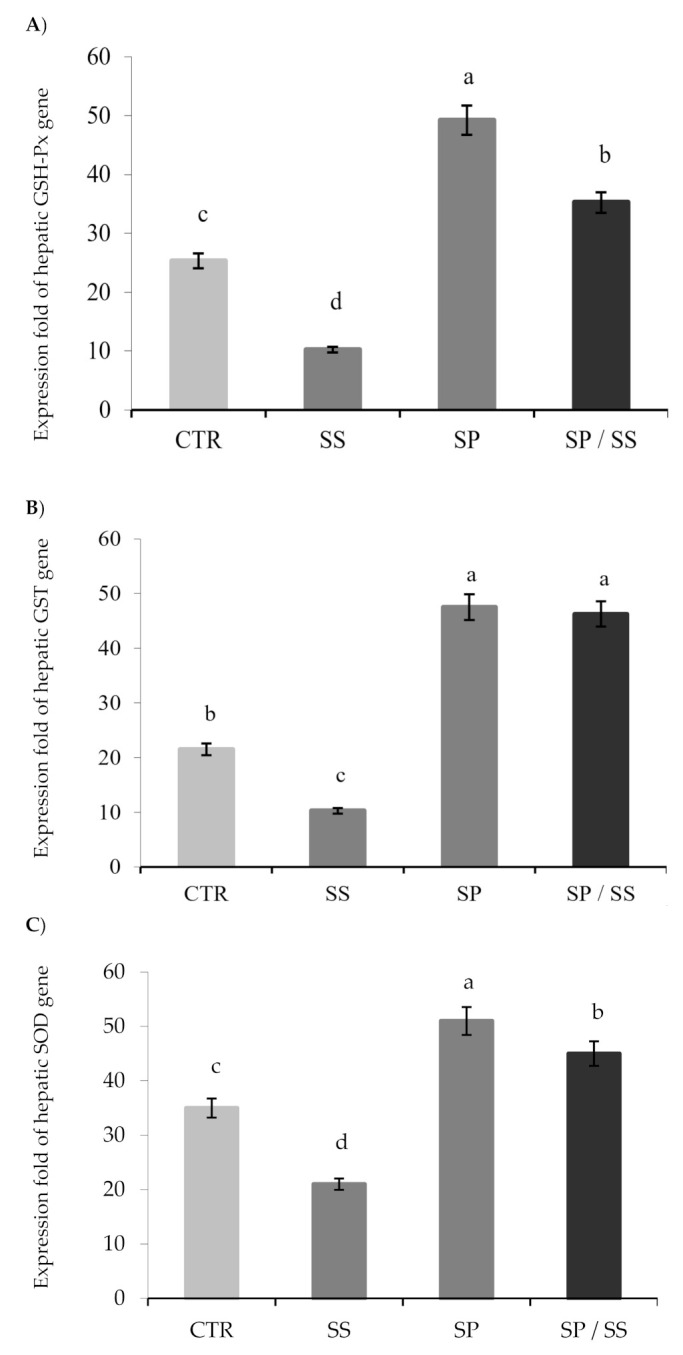
Relative expression of the hepatic antioxidant genes (**A**) GSH-Px, (**B**) GST, and (**C**) SOD in Nile tilapia after sodium sulphate (SS) exposure and fed diets with or without *S. platensis* (SP) for 8 weeks. a,b,c,d = letters on top of the bars show that the groups are significantly different from those in the control group (CTR) (*p* < 0.05).

Figure 4 illustrates the expression folds of the examined hepatic antioxidant genes, whereas there was significant downregulation of GSH-Px (Figure 4A), GST (Figure 4B), and SOD (Figure 4C) genes in the SS group (*p* < 0.05). Dietary SP significantly upregulated the expression of the hepatic antioxidant genes (*p* < 0.05). From the pairwise comparisons with the SS group, there was significant upregulation of GSH-Px, GST, and SOD genes (*p* < 0.05) in the group fed the diet supplemented with SP and consequently exposed to SS toxicity.

## 4. Discussion

Recently, there is continuous and increasing attention among researchers on finding safe feed supplements for potential attenuation of the undesirable and toxic influences of chemical pollutants on the exposed aquatic organism [36,37,38]. Considering the toxic impacts of SS on aquatic organisms’ performances [4], there should be a practical solution to counteract the oxidative stress induced by SS toxicity. The present study first examined the antioxidative responses of Nile tilapia fed dietary SP, which has an influence against oxidative stresses [16], to overcome SS-induced oxidative stress.

The growth performance of tilapia under the current trial conditions showed non-significant differences among the groups fed dietary *S. platensis* (SP) with or without sodium sulphate (SS) toxicity. Retention of growth performance with the SP diet even under SS exposure can probably be attributed to SP’s influence as a natural growth promotor [39]. Besides, dietary SP improved the antioxidation capacity of Nile tilapia exposed to sub-chronic toxicity of SS. The gills, muscle, liver, and kidney tissues of fish fed dietary SP had enhanced antioxidative responses with or without SS toxicity. Additionally, the antioxidative-related genes revealed upregulated expression by dietary SS.

Oxidative stress is a state in which there is abnormal overproduction of ROS to certain levels that overcomes the body endogenous shielding mechanisms and subsequently causes serious damage to the cellular components, including proteins, lipids, and nucleic acid [40]. It produces a condition of inequality between the production and elimination of ROS, which in turn helps the ROS to attack the cellular components, triggering lipid peroxidation (LPO) of the cell membrane, DNA damage, mitochondrial dysfunction, and finally cell death [41]. To maintain the redox homeostasis, the body triggers various scenarios to neutralize ROS, which can be classified into (1) enzymatic mechanisms, such as SOD, GST, CAT, and GSH-Px; and (2) non-enzymatic mechanisms, such as reduced GSH [42]. In the present study, oxidative stress was observed in the muscles, gills, liver, and kidney of tilapia exposed to SS, but fish fed SP showed a highly antioxidative response. The toxicity with SS causes overproduction of ROS, which impaired these tissues, and the body lost its ability to resist the oxidation.

The present study elucidated the possible roles of dietary SP in the mitigation of SS-induced oxidative stress in Nile tilapia, where fish were fed a basal diet supplemented with 1% SP and consequently exposed to SS sub-chronic toxicity for eight weeks. The results of this study exemplified a substantial diminution of SS-induced oxidative stress, whereas there was a pronounced increment of tissue GSH-Px, and CAT, SOD activities as well as an improvement of the T-AOC in the hepatic and renal tissues. Moreover, there was considerable upregulation of GSH-Px, GST, and SOD genes. Atli and Canli [43] reported similar oxidative stress following exposure of Nile tilapia to acute and chronic metal toxicity. Parallel to our findings, it was found that SP can mitigate the oxidative stress markers induced after exposure of Nile tilapia to chlorpyrifos [26], arsenic toxicity [19], and sodium sulphate toxicity [5].

The antioxidant function of SP can be attributed to its content of phycocyanin and carotenoids [44,45], and its high content of β-carotene, which helps to i) neutralize ROS molecules, such as hydroxyl, alkoxyl, and peroxyl radicals; and ii) reduce nitrite and nitric oxide (NO) synthase to diminish the hepatic LPO [46,47]. Despite the lack of vitamin C in the present study, which is also known for its antioxidative impact on the aquatic organisms [48], *S. platensis* is recognized as a rich source for vitamin C (~10.1 mg/100 g) and vitamin E (~5 mg/100 g) [49], which supports the antioxidative response of tilapia under the current trial conditions.

Toxicity with sodium sulphate can be detoxified by conjugation with glutathione (GSH-Px) during the catalysis of GST [50]. As the GST activity reduces, the conjugation of reduced GSH to various substrates would also be reduced. This activity may result in an increase of the GSH concentration in specific organs and the whole body. It was known that GST, in addition to its catalytic function, may directly bind to xenobiotic toxins [51]. This process helps reduce toxin concentrations in the body. Hence, the increased GST activity may indicate an improved defense system of the fish body. Interestingly, the results showed that the liver GST activity was increased by the SP concentration. Therefore, a reduction in GST may lead to toxic or even carcinogenic effects in fish. However, a detailed correlation between GSH-Px and GST activity, as well as the mechanism by which SP affect these enzymes, requires further study.

Like upregulated GST, the CAT level was increased in the gills of Nile tilapia fed SP. CAT is an antioxidant enzyme present in most organisms to catalyze hydrogen peroxide decomposition into water and oxygen [52]. Therefore, the upregulated GST and improved CAT are also considered one of the defense enzymes of Nile tilapia that are attributed to the potential role of SP as a natural antioxidant [13].

It is well noted that the oxidative stress induced by toxicity with pesticides and insecticides is also responsible for genotoxicity, inflammation, and immunosuppression in the immune-related cells of fish [11,53,54]. The present study showed damaged antioxidative-related genes in tilapia tissues by SS toxicity, but the results lack the detection of the immunity, inflammatory, and pro-inflammatory-associated genes, which may explain the regulatory role of SP as a natural anti-inflammatory supplement. Therefore, future studies are required to exemplify the potential role of SP as a natural antioxidant, immunostimulant, and anti-inflammatory supplement for the development of Nile tilapia farming.

## 5. Conclusions

Dietary *S. platensis* improved the antioxidation capacity of Nile tilapia exposed to sub-chronic toxicity of sodium sulphate. The gills, muscle, liver, and kidney tissues of Nile tilapia fed dietary *S. platensis* showed enhanced antioxidative responses with or without sodium sulphate toxicity. Concurrently, antioxidative-related genes revealed upregulated expression in tilapia fed dietary *S. platensis*. The enhanced antioxidative capacity of tilapia illustrated that dietary *S. platensis* is recommended to counteract the oxidation induced by sodium sulphate toxicity. Moreover, the regular inclusion of *S. platensis* in aquafeeds seems to be a beneficial biological approach to diminish the oxidative stress induced from the exposure of fish to toxic agents.

## Figures and Tables

**Table 1:** The ingredients and proximate chemical analysis (%) based on the dry matter (DM) basis of the basal diet used in the current study.

Ingredients	% on a DM Basis
Yellow corn meal	28.0
Fish meal	25.5
Soyabean meal	21.5
Wheat bran	9.5
Corn gluten meal	2.0
Rice bran	7.3
Gelatin	2.0
Fish oil	3.0
Mineral mixture ^a^	0.5
Vitamin premix ^b^	0.5
Di-calcium phosphate	0.2
**Chemical Proximate Analysis**	
Crude protein	31.78%
Crude fiber	5.66%
Ether extract	7.15%
Ash	8.14%
Gross energy (GE) ^c^	18.46 kcal/g

^a^ Mineral mixture (mg/kg diet) contains of the following: 40 iron (Fe), 80 manganese (Mn), 4 copper (Cu), 50 zinc (Zn), 0.5 iodine (I), 0.2 cobalt (Co), and 0.2 selenium (Se). Other primary minerals, such as magnesium (Mg), are expected to be provided from feed materials, e.g., fish meal. ^b^ Vitamins premix contains of the following: 500 mg/kg Vit. C, 5000 IU Vit. A, 2000 mg/kg Vit. E, 2000 mg/kg Vit. B_1_, 1000 IU Vit. D_3_, 20 mg/kg Vit. B_12_, 200 mg/kg Vit. K_3_, 500 mg/kg Vit. B_2_, 410 mg/kg Vit. B_6_, 1000 mg/kg Pantothenic acid, 1 mg/kg Folic acid, 1500 mg/kg Biotin, and 30 mg/kg Niacin (AGRI-VET for manufacturing Vitamins and Feed Additives, Tenth of Ramadan City A2, Egypt). ^c^ GE was calculated on basis of the values for protein, lipids, and carbohydrate as 23.6, 39.5, and 17.2 kJ/g, respectively.

**Table 2:** Primer sequences of antioxidant genes used in qRT-PCR analysis in the present study.

Genes	Accession No.	Sequence Primers	References
*SOD*	JF801727.1	Forward: 5′-CTCCAGCCTGCCCTCAA-3′	[31]
		Reverse: 5′-TCCAGAAGATGGTGTGGTTAATGTG-3′	
*GST*	EU234530.1	Forward: 5′-TAATGGGAGAGGGAAGATGG-3′	[33]
		Reverse: 5′-CTCTGCGATGTAATTCAGGA-3′	
*GSH-Px*	NM_001279711.1	Forward: 5′-CGCCGAAGGTCTCGTTATT-3′	[32]
		Reverse: 5′-TCCCTGGACGGACACTT-3′	
*β-actin*	EU887951.1	Forward: 5′-CAATGAGAGGTTCCGTTGC-3′	[34]
		Reverse: 5′-AGGATTCCATACCAAGGAAGG-3′	

*SOD*: Superoxide dismutase, *GST*: Glutathione-S-transferase, *GSH-Px*: Glutathione peroxidase, *β-actin*: Beta actin.

**Table 3:** The growth performance of Nile tilapia fed dietary *Spirulina platensis* (SP) with or without sodium sulphate (SS) toxicity.

Groups	IBW (g)	FBW (g)	WG (%)	SGR (%/Day)
Control	40.38 ± 0.01	83.75 ± 4.94	107.42 ± 12.08	1.21 ± 0.10
SS	40.47 ± 0.51	84.25 ± 3.28	108.25 ± 8.99	1.22 ± 0.07
SP	40.21 ± 0.22	85.00 ± 5.28	111.52 ± 14.04	1.24 ± 0.11
SP/SS	40.19 ± 0.34	83.25 ± 1.37	107.15 ± 3.02	1.21 ± 0.02

Values are expressed as mean ± standard errors (SE) (*n* = 3). The absence of letters indicates that feeding with dietary SP with or without SS toxicity had no effect on the growth performance of Nile tilapia (*p* > 0.05). IBW: initial body weight; FBW: final body weight; WG: weight gain; SGR: specific growth rate.
